# Surgical repair of partial anomalous pulmonary venous connection shunting from left atrium to innominate vein

**DOI:** 10.1186/1749-8090-8-100

**Published:** 2013-04-18

**Authors:** Dmitry Bobylev, Thomas Breymann, Dietmar Boethig, Masamichi Ono

**Affiliations:** 1 Department of Cardiothoracic, Transplantation, and Vascular Surgery, Hannover Medical School, Carl-Neuberg-Str. 1, 30625, Hannover, Germany; 2 Department of Pediatric Cardiology and Intensive Care Medicine, Hannover Medical School, Carl-Neuberg-Str. 1, 30625, Hannover, Germany

**Keywords:** Partial anomalous pulmonary venous drainage, PLSVC, Intact atrial septum

## Abstract

Partial anomalous pulmonary venous connection (PAPVC) causes a left-to-right shunt from the anomalous pulmonary vein (PV) to a systemic vein. We report an uncommon adult case of PAPVC, in which the left upper PV drained into both the innominate vein and the left atrium (LA), demonstrating retrograde shunting from the LA to the innominate vein. The anomaly was surgically repaired.

## Background

Partial anomalous pulmonary venous connection (PAPVC) of the left pulmonary veins is significantly less frequent than right ones, and most commonly drained to the innominate vein. However, there are many patterns of anomalous draining, causing shunt from left atrium (LA) to systemic veins [1].

## Case presentation

**Figure 1:**
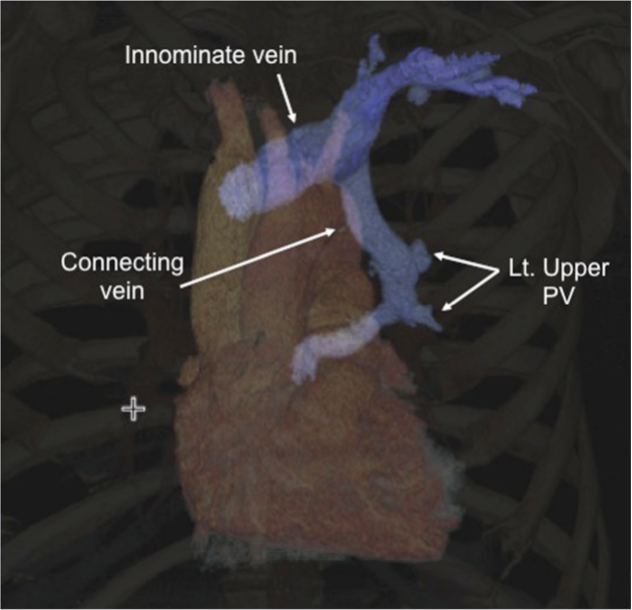
**Preoperative computed tomography image.** Three-dimensional CT showed left upper pulmonary vein connecting simultaneously to the innominate vein and the left atrium. PV, pulmonary vein.

**Figure 2:**
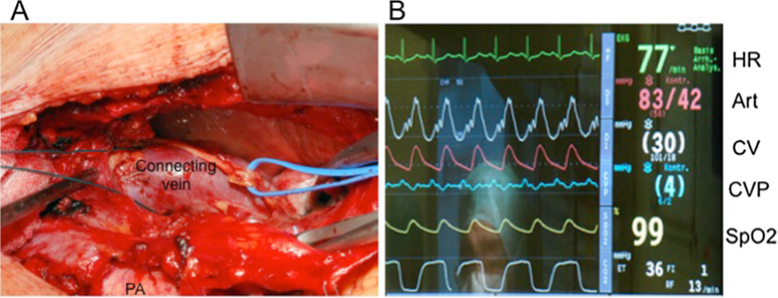
**Intra-operative photos. A**: The dilated connecting vein, between left upper pulmonary vein and the innominate vein, was identified. **B**: After ligation of the distal end of the connecting vein, marked pressure elevation was observed. HR, heart rate; Art, arterial pressure; CV, Connecting vein pressure; CVP, central venous pressure; SpO2, percutaneous oxygen saturation.

A 60-year-old man was admitted to our hospital under the diagnosis of PAPVC of the left upper pulmonary vein. He had progressive dyspnea of 1 year, and worsening shortness of breath, and increasing lower-extremity edema. Transthoracic and trans-esophagial echocardiogram revealed a moderately dilated right ventricle and normal left ventricle, no evidence of intracardiac shunt. Color-Doppler echocardiogram demonstrated retrograde blood flow from LA to innominate vein. To clarify the exact anatomy, a thorax CT was performed, which revealed the left upper vein draining both into the LA and the innominate vein (Figure 1). A cardiac catheterization showed 22 mmHg of mean pulmonary artery pressure and moderate left-to-right shunt (pulmonary/systemic flow ratio 2.9). His coronary angiogram showed no obstructive lesions. The elective operation was performed thorough median sternotomy. Firstly, the connecting vein between the left upper pulmonary vein and the innominate vein was identified (Figure 2A). Then, the distal end of the connecting vein was ligated and the pressure of the left upper pulmonary vein was measured. Immediately, marked elevation of the mean pulmonary vein pressure (30 mmHg) was noticed (Figure 2B). Then, cardiopulmonary bypass was initiated, rerouting of the connecting vein to the left atrial appendage was performed with cardioplegic cardiac arrest. The postoperative course was uneventful, and his symptoms were improved postoperatively. In a telephone follow-up 6 months after surgery with patient, he reported about no episodes of dyspnea or lower -extremity edema.

## Discussion

In the presented case, we describe an uncommon case of PAPVC of the left upper pulmonary vein, which drained both into the innominate vein and into the LA, causing left-to right shunt from LA to innominate vein. Snellen, et al. [1] described the patterns of anomalous pulmonary venous drainage using 52 autopsy cases and the 72 surgical cases, and showed one case of this anomaly. There are several case reports showing the similar pattern to our case [2-4]. We previously reported a case of partial anomalous pulmonary venous connection of the right upper pulmonary vein shunting from left atrium to superior vena cava [5], and in this case the division of the anomalous vein to the SVC was successfully done as a surgical repair. We had expected that simple ligation of the anomalous vein was enough to repair, but we needed the rerouting procedure to keep enough pathways from left upper pulmonary vein to the left atrium in the presenting case.

## Conclusion

In summary, we reported a rare adult case of PAPVC of the left upper pulmonary vein, which flow both into the innominate vein and the left atrium, requiring surgical rerouting of the distal end of the connecting vein to the left atrial appendage.

## Consent

Written informed consent was obtained from the patient for publication of this case report and accompanying images. A copy of the written consent is available for review by the Editor-in-Chief of this journal.

## Competing interests

The authors declare that they have no competing interests.

## Authors’ contributions

All authors have no financial or other interests regarding the submitted manuscript. DB conceived the study, provided the information of the patient, performed literature search, wrote and reviewed the manuscript, TB was the operating surgeon of the patient and participated in the coordination of this study, DB participated in drafting the manuscript, supervised and reviewed the manuscript, MO participated in drafting the manuscript, supervised and reviewed the manuscript. After critical revision of the resulting manuscript by MO, all authors have read and approved the final manuscript.
